# Supplementary material for: End Sequence Analysis Toolkit (ESAT) expands the extractable information from single-cell RNA-seq data
Source: Genome Res. 2016 Oct;26(10):1397–410. doi: 10.1101/gr.207902.116 (PMC5052061; doi:10.1101/gr.207902.116)
Supplement: Supplemental Material [file supp_gr.207902.116_Supp_Materials.tar.gz › ESAT-esat_v0.1/doc/html/classumms_1_1core_1_1readers_1_1_paired_end_reader.html]

ESAT: umms.core.readers.PairedEndReader Class Reference


ESAT


- Main Page
- Related Pages
- Packages
- Classes
- Files

- Class List
- Class Index
- Class Hierarchy
- Class Members

All Classes Namespaces Files Functions Variables Enumerator Pages

- umms
- core
- readers
- PairedEndReader

Classes |
Public Member Functions |
Static Public Member Functions |
List of all members

umms.core.readers.PairedEndReader Class Reference

|  |  |
| --- | --- |
| Classes | |
| enum | **AlignmentType** |
|  | |

|  |  |
| --- | --- |
| Public Member Functions | |
|  | PairedEndReader (File bam) |
|  | |
|  | PairedEndReader (File bam, TranscriptionRead read) |
|  | |
|  | PairedEndReader (File bam, TranscriptionRead read, boolean fra) |
|  | |
| AlignmentType | getAlignmentType () |
|  | |
| void | setFragmentFlag (boolean fra) |
|  | |
| void | close () throws IOException |
|  | |
| SAMFileHeader | getHeader () |
|  | |
| Set< String > | getSequenceNames () |
|  | |
| boolean | hasIndex () |
|  | |
| CloseableIterator< Alignment > | iterator () |
|  | |
| CloseableIterator< Alignment > | query (Annotation a, boolean contained) |
|  | |
| int | getRefSequenceLengths (String chr) |
|  | |
| Map< String, Integer > | getRefSequenceLengths () |
|  | |

|  |  |
| --- | --- |
| Static Public Member Functions | |
| static AlignmentType | getAlignmentType (SAMFileHeader header) |
|  | |
| static boolean | isPairedEndFormat (String fileToCheck) |
|  | |
| static String | getPairedEndFile (String fileToCheck) |
|  | |
| static String | getOrCreatePairedEndFile (String fileToCheck, TranscriptionRead txnRead) |
|  | |
| static void | main (String[] args) |
|  | |

## Constructor & Destructor Documentation

|  |  |  |  |  |  |
| --- | --- | --- | --- | --- | --- |
| umms.core.readers.PairedEndReader.PairedEndReader | ( | File | *bam* | ) |  |

|  |  |  |  |
| --- | --- | --- | --- |
| umms.core.readers.PairedEndReader.PairedEndReader | ( | File | *bam*, |
|  |  | TranscriptionRead | *read* |
|  | ) |  |  |

|  |  |  |  |
| --- | --- | --- | --- |
| umms.core.readers.PairedEndReader.PairedEndReader | ( | File | *bam*, |
|  |  | TranscriptionRead | *read*, |
|  |  | boolean | *fra* |
|  | ) |  |  |

Here is the call graph for this function:

## Member Function Documentation

|  |  |  |  |  |
| --- | --- | --- | --- | --- |
| void umms.core.readers.PairedEndReader.close | ( |  | ) | throws IOException |

|  |  |  |  |  |  |
| --- | --- | --- | --- | --- | --- |
| static AlignmentType umms.core.readers.PairedEndReader.getAlignmentType | ( | SAMFileHeader | *header* | ) |  |

static

Here is the call graph for this function:

|  |  |  |  |  |
| --- | --- | --- | --- | --- |
| AlignmentType umms.core.readers.PairedEndReader.getAlignmentType | ( |  | ) |  |

Here is the caller graph for this function:

|  |  |  |  |  |
| --- | --- | --- | --- | --- |
| SAMFileHeader umms.core.readers.PairedEndReader.getHeader | ( |  | ) |  |

Here is the caller graph for this function:

|  |  |  |  |
| --- | --- | --- | --- |
| static String umms.core.readers.PairedEndReader.getOrCreatePairedEndFile | ( | String | *fileToCheck*, |
|  |  | TranscriptionRead | *txnRead* |
|  | ) |  |  |

static

Finds a paired end file or creates one if it doesn't exist

Parameters
:   |  |  |
    | --- | --- |
    | fileToCheck |  |

Returns

Here is the caller graph for this function:

|  |  |  |  |  |  |
| --- | --- | --- | --- | --- | --- |
| static String umms.core.readers.PairedEndReader.getPairedEndFile | ( | String | *fileToCheck* | ) |  |

static

Returns the file itself or the file plus the default

PAIRED\_END\_EXTENSION

if either is in paired end format. If neither is in paired end format, returns null.

Parameters
:   |  |  |
    | --- | --- |
    | fileToCheck |  |

Returns

Here is the call graph for this function:

Here is the caller graph for this function:

|  |  |  |  |  |  |
| --- | --- | --- | --- | --- | --- |
| int umms.core.readers.PairedEndReader.getRefSequenceLengths | ( | String | *chr* | ) |  |

Get the lengths of the reference sequences

Parameters
:   |  |  |
    | --- | --- |
    | chr | the chromsoome to query size |

Returns
:   Map associating each reference name with sequence length

Here is the call graph for this function:

Here is the caller graph for this function:

|  |  |  |  |  |
| --- | --- | --- | --- | --- |
| Map<String, Integer> umms.core.readers.PairedEndReader.getRefSequenceLengths | ( |  | ) |  |

Get the lengths of the reference sequences

Returns
:   Map associating each reference name with sequence length

Here is the call graph for this function:

|  |  |  |  |  |
| --- | --- | --- | --- | --- |
| Set<String> umms.core.readers.PairedEndReader.getSequenceNames | ( |  | ) |  |

Here is the call graph for this function:

Here is the caller graph for this function:

|  |  |  |  |  |
| --- | --- | --- | --- | --- |
| boolean umms.core.readers.PairedEndReader.hasIndex | ( |  | ) |  |

|  |  |  |  |  |  |
| --- | --- | --- | --- | --- | --- |
| static boolean umms.core.readers.PairedEndReader.isPairedEndFormat | ( | String | *fileToCheck* | ) |  |

static

Returns

true

if the given SAM file is in paired end format (has the mateLine) attribute. Will also check the file with the default extension.

Parameters
:   |  |  |
    | --- | --- |
    | fileToCheck |  |

Returns

Here is the call graph for this function:

Here is the caller graph for this function:

|  |  |  |  |  |
| --- | --- | --- | --- | --- |
| CloseableIterator<Alignment> umms.core.readers.PairedEndReader.iterator | ( |  | ) |  |

|  |  |  |  |  |  |
| --- | --- | --- | --- | --- | --- |
| static void umms.core.readers.PairedEndReader.main | ( | String[] | *args* | ) |  |

static

Here is the call graph for this function:

|  |  |  |  |
| --- | --- | --- | --- |
| CloseableIterator<Alignment> umms.core.readers.PairedEndReader.query | ( | Annotation | *a*, |
|  |  | boolean | *contained* |
|  | ) |  |  |

Here is the call graph for this function:

|  |  |  |  |  |  |
| --- | --- | --- | --- | --- | --- |
| void umms.core.readers.PairedEndReader.setFragmentFlag | ( | boolean | *fra* | ) |  |

Here is the caller graph for this function:

---

The documentation for this class was generated from the following file:

- java/umms/core/readers/PairedEndReader.java


---

Generated on Fri Aug 15 2014 15:11:14 for ESAT by  

 1.8.7
